# A quantitative assessment of Chinese county high school policies using the PMC index model

**DOI:** 10.1371/journal.pone.0361807

**Published:** 2026-11-02

**Authors:** Qiulin Wu, Guanghai Li

**Affiliations:** 1 College of Educational Science, Northwest Normal University, Lanzhou, Gansu, China; 2 College of Education Science, Guangxi Normal University, Guilin, Guangxi, China; National Center for Chronic and Noncommunicable Disease Control and Prevention, Chinese Center for Disease Control and Prevention, CHINA

## Abstract

The county high school policies (CHSPs) is crucial for improving the quality of basic education in China’s counties and promoting rural social development. As policy documents are the foundational elements of policy systems, it is crucial to evaluate current policy texts, identify their shortcomings, and provide empirical evidence for future policy development. This study collected 55 policy texts from provincial and municipal governments, utilizing the Policy Modeling Consistency index model to evaluate 14 representative policies. The results indicate that all the assessed policies fall within the“pass”,“good”,or“with distinction” score category, with no substandard policies identified and none achieving a “perfect” score.A detailed analysis identifies critical policy shortcomings, including the prioritization of short-to-medium-term strategies over long-term planning, fragmented enrollment systems, insufficient campus culture development, as well as limited career guidance, mental health support,and policy guidance for home-school-community joint education. Local governments adopt a conservative stance on educational policy evaluation and systemic institutional reforms.To address these issues, this study recommends that local governments integrate the enrollment systems of public and private high schools within their jurisdictions, strengthen the prohibition of on cross-regional enrollment, increase investment in students’ career guidance and mental health education, and launch more programs to encourage parents and communities to participate in school education. Moreover, the central government should urge local authorities to accelerate reforms in the high school education system.

## 1. Introduction

In China, county high schools are senior high schools run by county-level governments, which provide general education. They have the highest educational quality and accommodate the largest student population at local level. Normally, there are one to three of these schools in an ordinary county, while in county-level cities with larger economic aggregate and population size, their number may be higher.

County high schools are the highest level of basic education at the county area in China; therefore, the quality of running schools is an important indicator of the level of local county education [1]. According to the Ministry of Education, in 2024 high school enrollment in county, town, and rural-level areas reached 13,175,995 students, 45% of the national total high school enrollment [2], the number of high schools in these areas reached 6,426 institutions, constituting 40% of all high schools nationwide [3]. Furthermore, county high schools are the most important element of the county-level education system. As the final stage of basic education in counties, they provide the only pathway for local students to enter universities. At the same time, they serve as an intermediate link in the education system, bridging basic education and higher education [4]. Accordingly, because of the number of rural students they serve and their institutional position in China’s education system, their teaching quality is directly related to educational equity between urban and rural areas in China.

In the past, county high schools enjoyed a fairly long period of prosperity, as a direct result of China’s basic education policy. In 1953, the central government proposed the establishment of 194 national key high schools [5]. Later on in 1994, the State Council of China promulgated relevant policies, requiring each county to run one or two well-developed middle schools [6]. Accordingly, one to two county high schools have been established in each county, representing the highest educational level and acquiring a key position in the county basic education system. Therefore, the rapid development of county high schools over a long period of time is largely attributable to the key high school policy. Although China revised the Compulsory Education Law of 2006 and abolished the classification schools into key schools and non-key schools, key senior high schools still exist in practice, due to historical inertia and the Matthew effect [7].

In recent years, a phenomenon of county high school decline, characterized by a persistent brain drain of high-quality teachers/students from county high schools and a continuous decline in the admission rates to universities of village students, has been frequently reported by media outlets nationwide, sparking widespread concern. One major cause of this problem is that a number of key high schools thriving under the key high school policy have received investments from privacy capital; this, coupled with their public-owned nature and deliberate policy guidance from the government, leads to their gradual evolution into “super high schools” [8]. These schools not only take advantage of their superior resources and brand influence to attract teachers from county high schools, but also recruit high-achieving junior high school students from county areas by offering substantial bonuses, leading to a massive loss of teachers and student resources in county high schools [9]. Furthermore, the lack of regulation over student mobility has exacerbated this problem [10].

In order to solve this problem, in December 2021 the Ministry of Education, together with nine other Departments, issued the *“14*^*th*^ *Five-Year Plan” for the Promotion of County High Schools*(hereinafter referred to as “14^th^ Five-Year Plan”), which is the first guiding document focusing on the county level [11]. This Plan aims to ensure that the educational quality of county high schools meets the requirements of equitable access to basic public education put forward in the outline of the *14*^*th*^ *Five-Year Plan for National Economic and Social Development and Long-Range Objectives for 2035* [12]. To further consolidate this goal, the *“Plan for Building China into a Leading Country in Education (2024-2025)”* clearly set the objective of “boosting the development of senior high schools in county-level areas” [13]. On the one hand, these policies shows that the existing problems of county high schools make them the “weak link” of the basic education at the county level, which does not align to the national requirements for urban-rural integration and development and for new urbanization construction [1], showing the need for continuous efforts and the continuous attention of the academic community. On the other hand, with the end of the 14^th^ Five-Year Plan and the development of the following one, the problems of the current county policy and the expected direction of future interventions have increased the need for targeted county policy research. Therefore, this study targets county high schools policies(CHSPs) issued by local governments as its research subjects.

This study addresses the following issues:

- What is the quality of the CHSPs currently issued by local governments in China?
- Which specific issues exist in the CHSPs formulated by local governments?
- How can these CHSPs be improved?

The main contributions of this study are as follows: Firstly, it employed text mining techniques to conduct an in-depth analysis of China’s county high school policies, evaluating their content and providing novel perspectives for related research. Secondly, it introduced a methodological innovation to existing research, establishing a quantitative evaluation framework for Chinese CHSPs based on the policy modeling consistency (PMC) index model. Thirdly, it selected 55 policy documents as research samples and conducted a quantitative assessment of 14 representative policies, generating new empirical evidence to inform adjustments to China’s CHSPs.

This paper is structured as follows: Section 2 reviews the pertinent literature; Section 3 outlines the research design; Section 4 details the construction of the evaluation index system; Section 5 describes the policy assessment process; Section 6 presents the calculation results of the PMC index and generates surface plots; Section 7 provides a detailed analysis of the research findings; and Sections 8 and 9 outline the conclusions and policy proposal, respectively.

## 2. Literature review

### 2.1. Existing research on CHSPs

Existing research on the widespread operational difficulties and declining educational quality in China’s county high schools, can be broadly grouped into three categories. The first category of studies adopted the analytical lens of internal educational organizational structures, attributing the decline of county high schools to the unregulated expansion of regional key high schools and super high schools.For example, Yang and Wang argued that the industrialization and commercialization of super high schools undermine local basic education systems through cross-regional recruitment of top students and high-salary incentives to attract quality teachers [14]. The expansion of super high schools exacerbates inter-school disparities in educational resources, information, and reputation among regular high schools, thereby intensifying the Matthew effect between high schools [15], and significantly reducing the quality of education in all regular high schools [16].

The second category of studies posits that the decline of county high schools is the result of urbanization-driven population migration mechanisms. The migration of population to central cities leads to the concentration of rural teachers and students in urban areas, and the siphoning effect of super high schools exacerbates the decline of county high schools [17]. Moreover, prolonged urbanization has engendered a collective psychological expectation of urban educational superiority over rural counterparts in rural communities, thereby triggering an exodus from county high schools that ultimately result in the decline of these institutions through a self-fulfilling prophecy mechanism [18]. Even more disconcertingly, empirical evidence demonstrates the nationwide prevalence of this phenomenon [19].

The third category of studies examined the institutional determinants of county high school decline through the lens of educational governance and policy implementation mechanisms. These studies claim that, due to China’s fiscal decentralization framework, county-level governments bear full responsibility for educational funding, while being subject to two types of constraints: the finite fiscal capacity of local treasuries and the compulsory education expenditure mandates stipulated in Article 42 of the Compulsory Education Law of the People’s Republic of China. This structural contradiction systematically restricts fiscal allocations to county high schools, resulting in chronic underfunding of secondary education in rural jurisdictions [20]. In addition, quantitative research demonstrated that precisely due to the strong correlation between county-level education quality and local fiscal revenue capacity, the educational quality in China’s coastal county regions consistently surpasses that of inland areas [21]. Furthermore, scholars identified that a fundamental cause of subpar teaching quality in county high schools lies in unrestricted recruitment practices, through which elite urban institutions systematically recruit academically outstanding students from rural counterparts, thereby triggering a sustained brain drain that demoralizes teaching faculties [8]. In terms of policy analysis, some authors employed text coding and frequency analysis to examine policy priorities across 26 provincial-level CHSPs documents [22], offering useful methodological insights. However, these studies did not cover city-level policies. In fact, the idealized CHSPs framework must achieve two imperatives, i.e., universalization through comprehensive coverage and the diversification of educational provision, while enforcing rigorous controls to ensure that inter-regional disparities in teaching quality remain within an acceptable range [6]. These policy objectives encounter significant implementation challenges stemming from a three-dimensional institutional friction: an ambiguous delineation of governmental mandates, cognitive-resource mismatches with target beneficiary cohorts, and binding constraints within the policy ecosystem [23]. To solve these problems, Deng proposed a theoretical framework [24]. Other scholars employed sociological and political theoretical frameworks to assess the decline of county high schools, including elements such as cultural ecological reconstruction [25] and national educational governance [26].

These studies provided multidimensional perspectives to understand and address the decline of county high schools. However, it is noteworthy that they focused mainly on analyzing the causes of this decline and proposing remedial strategies, with limited attention directed toward the institutional design and operational mechanisms of CHSPs. This oversight has resulted in significant research gaps regarding systemic solutions, leaving future policy development inadequately supported by empirical evidence.

### 2.2. Policy content analyses

Recent years have witnessed significant advancements in quantitative policy text research, with a methodological diversification across three primary categories: policy metrology analysis, policy content quantification, and policy text mining. The analysis of policy content typically includes both a qualitative and a quantitative examination of policy texts [27]. Policy text quantification consists in the transformation of unstructured policy documents into abstract, feature-specific structured data through systematic conversion paradigms.

Conceptualized by Ruiz Estrada through the Omnia Mobilis theoretical lens, the PMC index modeling framework has distinct advantages over conventional text mining and mechanical element analysis methods. Traditional text mining methods are predominantly centered around word frequency statistics, and are primarily suited for thematic analysis and sentiment analysis of long texts [28,29]; however, they neglect the distinctive features of policy texts and are devoid of tools to assess, their quality. Simultaneously, mechanical element-based approaches depend on manually assigned element weights, which are intrinsically affected by the subjective factors of researchers. In this study, we chose the PMC index model as our main research method for the following reasons: (1)its parameter configuration based on policy text mining minimizes subjective human evaluation, thus substantially enhancing assessment objectivity and precision; (2)it deliberately avoids the hierarchical weighting of indicators, eliminating restrictions on variable quantity and significance, thereby enabling the comprehensive capture of potential influencing factors; (3)it facilitates cross-policy consistency analysis and visual representation of policy effectiveness, providing scientifically robust foundations for policy optimization; (4) its operational simplicity and cost-effectiveness enhance its practical applicability.

The PMC index model has gained increasing recognition in Chinese public policy research with extensive applications in sectors such as industrial policy [30–32], public health policy [33], energy policy [34], environmental policy [35],and public safety policy [36]. Previous empirical studies validated its effectiveness in large-scale public policy evaluation, demonstrating particular adaptability to educational policy analysis. Moreover, recent academic explorations have successfully implemented this model across diverse educational domains, including compulsory education policy [37] and higher education policy [38–40]. These applications collectively affirm the scientific validity and feasibility of application of this model in educational public policy research. Accordingly, this study employed the PMC index model to analyze the implementation of CHSPs by local government during the 14^th^ Five-Year Plan period.

## 3. Research design

This study performed a horizontal assessment of representative local policy documents of county high schools, with the aim to understand the policy focus of local governments and identify their deficiencies.

### 3.1. Data sources

This study used keywords such as “county-level high schools”, “county high schools” and “regular county high schools” to search the website of the Ministry of Education, the website of Chinese government, and the PKULAW database as supplementary and proofreading sources. The policy texts were selected for investigation based on the following requirements:

- They have been issued by provincial, city and county governments
- They consist in policy documents such as plans, opinions and notices; and
- Their content is highly related to the establishment of county high schools.

Policy texts whose topic is not related to the research subjects, as well as informal documents such as notifications, replies, and speeches were excluded from analysis.

Finally 55 documents were selected for variable setting and parameter identification, see S1 Appendix, issued in the period from December 1, 2021 to August 30, 2024.

### 3.2. Research method

This study employed three research methods. First, word frequency statistics were employed to identify the most important elements in policy texts and determine the specific elements of policy priorities and policy objects among the first-level indicators.

Second, text coding was performed using Grounded Theory(GT). Given the complexity of policy priorities and policy objects, due to the fact that multiple measures in policy texts may refer to a same element, while at the same time a single element may also cover various measures it was necessary to categorize these elements through text coding to construct a third-level indicator system.

Thirdly, this study employed the PMC index model, which allow a quantitative evaluation of policy texts after completing text preprocessing. Further details on these three methods are presented in the following subsections.

#### 3.2.1. Word frequency statistics.

Word frequency statistics are one of the common methods for policy text measurement, and is mainly used to display and describe the basic situation, the overall characteristics and the evolution trends of policy collections [41]. This method is also applied to the preprocessing of policy texts by relevant studies that adopted the PMC index model for policy evaluation [42].

In this study, word frequency statistics were performed on the 55 collected policy texts by calling Python’s Jieba segmentation module. The purpose of this approach was to intuitively identify the focus elements of these policy texts, which would serve as an important reference for the subsequent construction of the indicator system.

#### 3.2.2. Grounded theory (GT).

Firs proposed by Glaser and Strauss [43], GT is articulated in three iterative steps: Open Coding, Axial Coding and Selective Coding. During the whole process, researchers continuously compare, revise and integrate data with the concepts and categories established at the outset until theoretical saturation is achieved, i.e., until when newly collected data will no longer yield new attributes or insights for the established theoretical categories. This ensure that the theories generated by GT are rooted in abundant real-world data, boasting strong empirical relevance and explanatory power.

In this study, GT was adopted because the PMC index model requires the construction of an indicator system. However, as the the indicator table constructed by Estrada [29] could not be directly adopted, and customized indicator systems had to be built for different policy texts. Therefore, the indicator system was constructed based on the texts of the CHSPs, so that to provide a sufficient basis for each indicator and enhance their credibility.

To this purpose, the semantics in the original text were transformed into measurable indicators through the coding and induction of the original policy text.The indicators established in this way are mainly the tertiary indicators in policy priorities and policy objectives.

#### 3.2.3 The PMC index model.

First proposed by Mario Arturo Ruiz Estrada, the PMC index model is a classic tool for quantitative policy evaluation. Rooted in the *omnia mobilis* (everything is moving) assumption, it breaks through the limitation of the traditional ceteris paribus (all else being equal) assumption, enabling multi-dimensional and systematic assessment by incorporating all relevant variables into a unified analytical framework [28,29].

This model consists in converting unstructured policy texts into quantifiable values by constructing a multi-level evaluation indicator system that typically includes 9–10 first-level indicators (e.g., policy duration, nature, audience, focus, and function), each subdivided into several second-level indicators. A binary scoring method is adopted, assigning 1 for compliance with criteria and 0 otherwise; the final PMC index, calculated via weighted aggregation, reflects overall policy quality, with higher values indicating better policy performance.

## 4. The research process

Estrada’s PMC model is articulated in six steps: 1. Establish a multi-input-output table; 2. Confirm variables and specific parameters; 3. Calculate the PMC index; 4. Formulate consistency evaluation criteria for policy modeling; 5. Draw the PMC surface graph; 6. Analyze the advantages and disadvantages of policy modeling based on evaluation criteria [29].After reviewing relevant target literature, it was found that Chinese scholars followed five steps when applying this model policy text collection and preprocessing, indicator system construction, PMC index calculation, PMC surface graph drawing and comprehensive policy analysis [40].The specific process is shown in Fig 1.

**Fig 1:**
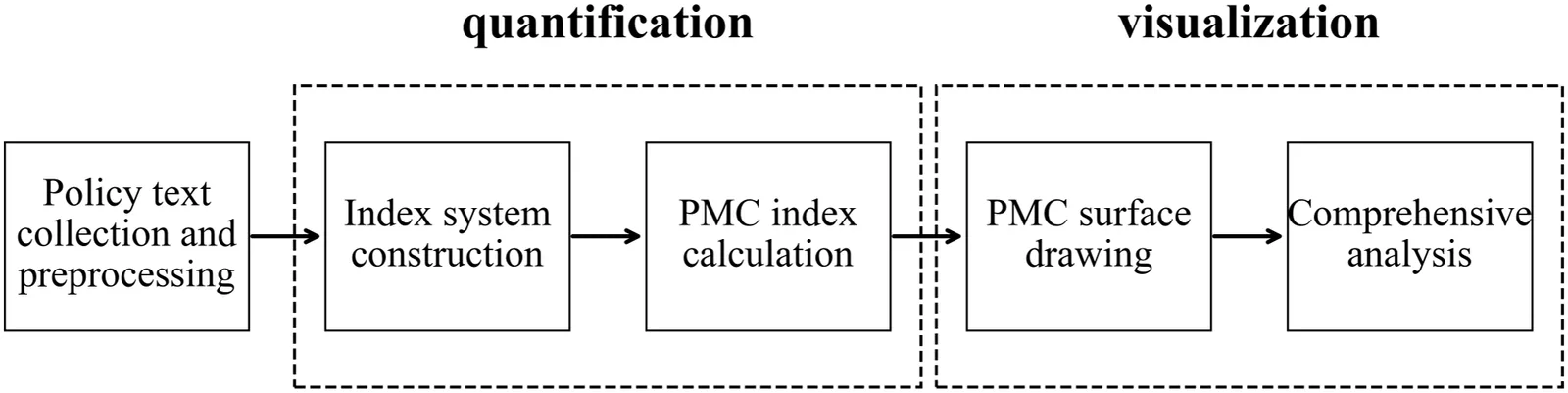
Theoretical framework of the PMC index model.

First, we had to collect and process the acquired texts. Among the various existing methods for preprocessing, we adopted word frequency statistics and GT. Next, based on the results of the preprocessing. Then, we established the indicator system for the PMC index model. Then we scored the selected policy texts as evaluation objects in accordance with the indicator system, after which, we calculated the PMC index using specific formulas and placed the results into a 3×3 matrix to generate a surface graph, which enabled an intuitive analysis of the strengths and weaknesses of each policy text.

### 4.1. First-level variable classification and parameter identification

Before constructing the PMC index model, the evaluation indicator system had to be established. First, in line with the 14^th^ Five-Year Plan,this study identified three primary first-level variables: policy objectives, policy priorities, and policy safeguards. These three indicators were established level 1 indicators. Second, the 55 compiled and screened policy texts were analyzed using Jieba for part-of-speech tagging and frequency statistics. Functionally insubstantial verbs, adjectives, and adverbs commonly found in policy documents were filtered out. This process yielded a ranked list of the top 50 keywords with the highest frequency of occurrence, presented in Table 1. It can be seen that“schools”, “teachers”, “principals” and “students” have a high frequency of occurrence these were classified as policy objects. High-frequency terms such as “model”, “supervision” and “fund” were categorized as policy guarantees. Finally, “school operations”, “teaching” and “student enrollment” were categorized as policy priorities.

**Table 1:** The 50 most frequently occurring terms in the analyzed policy.

Rank	Keyword	Frequency	Rank	Keyword	Frequency
1	County high schools	5575	26	County area	329
2	Education	1638	27	Synthesize	319
3	Develop	1566	28	Standard	303
4	School	1342	29	High quality	300
5	Teacher	1338	30	Support	290
6	Build	1017	31	Guide	288
7	Promote	989	32	Government	273
8	Student enrollment	966	33	Cultivate	271
9	School operations	670	34	Plan	257
10	Entrusted management assistance	484	35	Supervision	254
11	Put into effect	481	36	Strictly Forbid	247
12	Reform	474	37	College entrance examination	243
13	Principal	472	38	Coordinate	243
14	Manage	428	39	Instructional research	240
15	Teaching	409	40	Standardization	238
16	Department	393	41	Plan as a whole	236
17	Model	388	42	Country	235
18	Appraise	383	43	Improve	229
19	Assurance	372	44	Power	229
20	Student	372	45	Method	219
21	Funds	367	46	Regular	212
22	Duty	362	47	Organization	209
23	Characteristic	357	48	Running conditions	204
24	Horizontal	350	49	Large class	200
25	Quality	332	50	Condition	195

Note: All the words in this table were translated from Chinese.

### 4.2. Three-level variable classification and parameter identification

In order to accurately identify the second and third-level variables, this study coded the 55 policy documents investigated using NVivo 12 plus software transforming the original text into a conceptualized version through a systematic inductive analysis.

#### 4.2.1. Open coding.

Open coding consists in disassembling the original policy text, and identifying and tagging the potential concepts and categories as much as possible. In this study, the 55 policy texts were coded line by line, obtaining 2481 reference points in which 271 initial concepts were summarized. Next, the initial concepts with similar meanings were summarized, determining 48 initial categories, as shown in S2 Appendix.

#### 4.2.2. Axial coding.

Axial coding consists in analyzing and summarize the initial categories on the basis of open coding, identifying the main categories according to their internal logic and category. In this study, 48 initial categories were summarized and grouped into 14 main categories enrollment, teaching quality, teaching reform, entrusted management assistance, coordinated development, schools, teachers, principals, students, main responsibility, organization and leadership, financial support, publicity and guidance, supervision and assessment.

#### 4.2.3. Selective coding.

Selective coding consists in the further induction and differentiation of the axial coding. In this study, enrollment, teaching quality, teaching reform, entrusted management assistance, and coordinated development were identified as the policy priorities of local counties schools, teachers, principals and students were identified as policy objects and main responsibility, organization and leadership, financial support, publicity, and guidance, and supervision and assessment were identified as policy guarantees.

#### 4.2.4. Coding reliability.

In this study, all the three stages of open, axial, and selective coding were performed jointly by the two authors and a doctoral candidate specializing in educational policy. The corresponding author formulated the coding guidelines for the unified policy texts, while the first author and the doctoral candidate conducted independent line-by-line coding and category induction. To ensure coding consistency and objectivity, 10 policy documents were randomly selected for inter-coder reliability testing using the Cohen’s Kappa coefficient. After cross-checking and a consensus discussion on inconsistent coding entries, the final value of the Cohen’s Kappa coefficient reached 0.78, indicating a good level of inter-coder agreement. The unified coding standards and reliability control were consistently implemented throughout all the three coding procedures to guarantee the methodological rigor of the qualitative analysis.

#### 4.2.5. Saturation test.

A systematic saturation test after completing the coding of the original 55 CHSPs documents. Three additional policy texts were purposively sampled to represent distinct administrative levels: (1) a county-level comprehensive education improvement policy issued by the Debao County, Guangxi Zhuang Autonomous Region; (2) a provincial-level specialized high school development policy issued by Henan Province; and (3) a municipal-level medium-to-long-term education development plan issued by Huizhou City, Guangdong Province. The selected documents are: *“Notice of the People’s Government of Debao County on Printing and Distributing the Implementation Plan for Debao County to Implement the Three-Year Action Plan for Education Quality Improvement in Guangxi (2021-2023)”“Notice of the General Office of Henan Provincial Department of Education on Further Promoting the Diversified Development of Ordinary High Schools”* and *“Implementation Plan for Huizhou “Thousands of High-Quality Development Projects” in Education (2023–2027)*.” Notably, as these policies only partially address county high school development, only those text segments explicitly mentioning county high schools were coded for the saturation test. The authors applied the pre-established coding manual to these relevant text segments following the identical open, axial, and selective coding procedures used for the main sample. Coding discrepancies were resolved through consensus discussion. No new initial concepts, subcategories, main categories, or causal/associative relationships between categories were identified in any of the three documents. This confirmed that the theoretical framework developed in this study achieved full theoretical saturation, with no additional information requiring integration into the indicator system.

Based on the results of the word frequency statistics analysis, the GT analysis, Estrada [29] and relevant domestic research and the characteristics of local and county policies, and on relevant expert opinions, 9 first-level indicators, 35 second-level indicators and 48 third-level indicators were finally obtained, as shown in Table 2.

**Table 2:** Hierarchical structure of policy evaluation variables.

Level 1 variable	Level 2 variables	Level 3 variables	Reference
X1 Policy functions	X1:1 Directive role	—	Estrada [29]
	X1:2 Supporting role	—	
	X1:3 Encouragement effect	—	
	X1:4 Regulatory role	—	
X2 Policy statute of limitations	X2:1 Long-term (over 5 years)	—	Estrada [29]
	X2:2 Mid-term (3–5 years)	—	
	X2:3 Short-term (1–3 years)	—	
	X3:1 Students enrollment	X3:1–1 Integrated network system	
		X3:1–2 Enrollment regulation	
		X3:1–3 School-Based Quota Allocation	
		X3:1–4 Enrollment rate	
		X3:1–5 General-Vocational education integration	
	X3:2 Teaching quality	X3:2–1 Feature-based school development	
		X3:2–2 Informatization	
		X3:2–3 Dissemination of teaching outcomes	
		X3:2–4 Instructional research System	
		X3:2–5 Simultaneous development of the five types of educations	
	X3:3 Teaching reform	X3:3–1 Evaluation reform	
		X3:3–2 Teaching innovation	
		X3:3–3 Curriculum reform	
		X3:3–4 Optional class system	
X3 Policy priorities	X3:4 Entrusted management assistance	X3:4–1 Guidance and training	Word frequency statistics and GT
		X3:4–2 Paired assistance	
		X3:4–3 Flow of talents	
		X3:4–4 Legalization	
		X3:4–5 Fund guarantee	
		X3:4–6 Commendation and award	
	X3:5 Coordinated development	X3:5–1 Coordination between public and private schools	
		X3:5–2 General-vocational coordination	
		X3:5–3 Regional coordination	
	X4:1 School	X4:1–1 Layout optimization	
		X4:1–2 Standardization	
		X4:1–3 Reduction of oversized classes	
		X4:1–4 Information	
		X4:1–5 School-running funds	
		X4:1–6 Cultural construction	
		X4:1–7 Accommodation and health conditions	
		X4:1–8 Scale regulation	
		X4:1–9 School-running conditions	
		X4:1–10 Safety assurance	
	X4:2 Teacher	X4:2–1 Teaching staff replenishment	
		X4:2–2 Training	
		X4:2–3 teacher personnel management	
		X4:2–4 Teacher’s ethics	
		X4:2–5 Incentives	
X4 Policy object	X4:3 Principal	X4:3–1 Principal Team building	Word frequency statistics and GT
		X4:3–2 Competency training	
		X4:3–3 Employment regulations	
		X4:3–4 Incentive mechanism reform	
	X4:4 Students	X4:4–1 Educational expenditure per student	
		X4:4–2 Scholarships and grants	
		X4:4–3 Development guidance	
		X4:4–4 Personalized cultivation	
		X4:4–5 Comprehensive quality	
		X4:4–6 Collaborative education	
X5 Policy tools	X5:1 Command-type tool	—	McDonnell, Elmore [44] and Schneider, Ingram [45]
	X5:2 Incentive tool	—	
	X5:3 Capacity-building tool	—	
	X5:4 Exhortation tool	—	
	X5:5 System reform tool	—	
X6 Policy guarantee	X6:1 Principal responsibility	—	Kuang et al. [46]
	X6:2 Organization and leadership	—	
	X6:3 Financial support	—	
	X6:4 Publicity and guidance	—	
	X6:5 Supervision and assessment	—	
X7 Policy operability	X7:1 Specific scheme	—	Qian et al. [47]
	X7:2 Detailed measures	—	
	X7:3 Supporting policies	—	
	X7:4 Clear Division of Responsibilities	—	
X8 Policy evaluation	X8:1 Clear goals	—	Cui et al. [40]
	X8:2 Sufficient evidence	—	
	X8:3 Detailed planning	—	
	X8:4 Rational Strategy	—	
X9 File reference	X9:1 File reference	—	Estrada [29]

Note: In the variable of simultaneous development of the five types of educations (X3:2–5), these include moral, intellectual, physical, aesthetic, and labor education. X4:4–6 Collaborative Education refers to family-school-community collaborative education.

## 5. The policy evaluation process

### 5.1. Selection of policy texts

The selection of policy texts to enter the evaluation phase followed these criteria:

1. Selection of two cities or provinces from each of China’s seven major regions: Northeast China, North China, Central China, East China, South China, Southwest China, and Northwest China.
2. Selection of cities or provinces with a relatively developed economy and society, and a high population density.

These selected cities or provinces feature a high level of urbanization, as well as a more prominent imbalance in the distribution of educational resources between urban and rural areas and a serious decline of county high schools [19]. The final selected policy texts are shown in Table 3.

**Table 3:** List of CHSP texts.

Number	Title of the Policy Document	Release Date	Area
P1	Jilin Province “14^th^ Five-Year Plan” county-level high schools development and promotion action plan	2022	Northeast China
P2	Action Plan for the Development and Promotion of county-level high schools in Shenyang City (2023–2025)	2023	Northeast China
P3	Shanxi Province “14^th^ Five-Year Plan” county-level high schools development and promotion action plan	2021	North China
P4	Implementation plan for the development and promotion of county-level high schools in Hulunbuir city and county level	2023	North China
P5	Implementation plan of county-level high schools for the “14^th^ Five-Year Plan” Plan of Henan Province	2022	Central China
P6	Action Plan for the Development and Promotion of county-level high schools in Changsha City (2023–2025)	2023	Central China
P7	Action Plan for the development and promotion of county-level high schools in the “14^th^ Five-Year Plan” Plan period of Fujian Province	2022	Eastern China
P8	Action plan for quality improvement of ordinary high schools in 4 mountainous counties of Hangzhou	2023	Eastern China
P9	Action Plan for the Development and promotion of regular high schools in counties during the “14^th^ Five-Year Plan” period of Guangdong Province	2023	South China
P10	Nanning city “14^th^ five-year” county-level high schools development and promotion action plan implementation plan	2023	South China
P11	Opinions on the Implementation of the action plan for the Development and Promotion of county-level high schools in the “14^th^ Five-Year Plan” Period in Guizhou Province	2022	Southwest China
P12	Implementation plan of the action plan for development and promotion of regular county high schools during the “14^th^ Five-Year Plan” period in Chengdu	2023	Southwest China
P13	Gansu Province “14^th^ Five-Year Plan” county-level high schools development and promotion action plan	2022	Northwest China
P14	Development of county-level high schools in the “14^th^ Five-Year Plan” county seat of Ningxia Hui Autonomous Region	2022	Northwest China

Note: All the title in the table are translated from Chinese.

### 5.2. Establishment of a multi-input-output table

A multi-input-output table was constructed as shown in S2 Appendix, where the policy text was assigned according to a binary system, including two-level and three-level indicators. More in detail, a value of 1 was assigned to those policy texts proposing the relevant keywords or including a detailed description, and 0 otherwise as shown in S3 Appendix.

To mitigate subjective bias and ensure the objectivity of the scoring process, a standardized multi-rater scoring procedure was implemented following the development of the evaluation framework. A purposive sample of 7 graduate students was recruited based on their disciplinary expertise, including 2 students specializing in public policy and 5 students specializing on educational policy. All the raters received systematic training on the standardized scoring guidelines presented in Table 6, and completed a pilot scoring exercise to ensure a consistent understanding of the evaluation criteria. Each rater independently scored 2 distinct policy documents using the established rubric. As raters were not assigned the same policy texts, inter-rater reliability coefficients (e.g., Cohen’s Kappa) were not calculated. The scoring results were directly aggregated and cross-checked for consistency, and any ambiguous cases resolved through group discussion. The final aggregated scoring results are presented in S4 Appendix.

### 5.3. Calculation of the PMC index model

The PMC index model was calculated based on the scoring results presented in S4 Appendix, according to which the scores of the first-level variable for each policy document investigated were obtained. The calculation formulas are as follows:


$$\begin{array}{c} X\sim 𝒩(0,1) \end{array}$$ (1)

$$\begin{array}{c} X=X_{R}\in [0,1] \end{array}$$ (2)

$$\begin{array}{c} X_{m}=\left\{ \begin{array}{cc} \frac{1}{k}\sum_{n=1}^{k}X_{mn}, & m=1,2,5,6,7,8,9\\ \frac{1}{kl}\sum_{n=1}^{k}\sum_{l=1}^{L}X_{mnl}, & m=3,4 \end{array}\right. \end{array}$$ (3)

$$\begin{array}{cc} \text{PMC}= & \sum_{n=1}^{4}\frac{X_{1n}}{4}+X_{2}\sum_{n=1}^{2}\frac{X_{2n}}{2}+X_{3}\sum_{n=1}^{5}\sum_{l=1}^{L}\frac{X_{3nl}}{kl}\\ & +X_{4}\sum_{n=1}^{4}\sum_{l=1}^{L}\frac{X_{4nl}}{kl}+X_{5}\sum_{n=1}^{4}\frac{X_{5n}}{4}+X_{6}\sum_{n=1}^{5}\frac{X_{6n}}{2}\\ & +X_{7}\sum_{n=1}^{4}\frac{X_{7n}}{2}+X_{8}\sum_{n=1}^{4}\frac{X_{8n}}{2}+X_{9} \end{array}$$ (4)

The scores of all first-level variables were between [0,1]. Next, a sum was performed of these scores to calculate the PMC index. Finally, the 14 policies were evaluated following the criteria presented in Table 4 as modified by Estrada [29].

**Table 4:** Criteria for the evaluation of the policy PMC scores.

PMC score	0–5.99	6–6.99	7–7.99	8–8.99	9–10
Evaluation of the score	Fail	Pass	Good	With distinction	Perfect

Where, *m* is the ordinal number of first-level variables, textitm = 1, 2, 3, 4, 5, 6, 7, 8, 9. *n* is the ordinal number of second-level variables, ranging from 1 to *k* depending on the specific group. *l* is the ordinal number of third-level variables specifically for groups *m* = 3 and *m* = 4, ranging from 1 to *L*. *k* and *L* represent the total counts of second-level and third-level variables respectively in the corresponding analysis.

### 5.4. Building the PMC surface graph

A PMC surface graph was built based on the calculation results of the PMC index model, so as to intuitively present the advantages and disadvantages of the policy indicators. Specific the nine first-level indicators of the sample policy into a 3×3 matrix, as follow:


$$\begin{array}{c} \text{PMC surface}=\left\{\begin{array}{ccc} X_{1} & X_{2} & X_{3}\\ X_{4} & X_{5} & X_{6}\\ X_{7} & X_{8} & X_{9} \end{array}\right\} \end{array}$$ (5)

## 6. Computation results and scores visualization

Based on the computational results, the performance measurement and the comparison scores, as well as the corresponding evaluation grades for the 14 policies investigated were systematically performed; the results are shown in Table 5. The matrix of all PMC scores is presented in Table 6. For the mapping procedure Python MATLAB’s mesh grid function was employed to construct a 3D spatial grid, which served as the foundation for subsequent surface matrix fitting. Utilizing the V4 interpolation method, a smooth 3D surface map was generated, which effectively shows the complex relationships between policy attributes and performance outcomes. The 3D surface map presented in Fig 2 and Fig 3 shows 14 primary policy indicators convex areas indicate higher scores, while concave regions indicate lower scores, and darker tones indicate weaker outcomes. P14 showed an overall superior quality with localized deficiencies at X2 and X3, while P1 exhibited systemic underperformance except at X1, X6 and X9. The following Section presented the analysis of the policies from both a horizontal and a vertical perspective.

**Table 5:** PMC scores and evaluation grades of policy texts.

Metric Score	X1	X2	X3	X4	X5	X6	X7	X8	X9	PMC	Score Grade
P1	1.00	0.67	0.75	0.64	0.40	1.00	0.75	0.75	1.00	6.96	Pass
P2	1.00	0.33	0.79	0.81	0.60	1.00	1.00	1.00	1.00	7.53	Good
P3	1.00	0.67	0.84	0.83	0.80	1.00	0.75	1.00	1.00	7.88	Good
P4	1.00	0.33	0.96	0.76	0.60	0.75	1.00	0.75	1.00	7.16	Good
P5	1.00	0.67	0.69	0.63	1.00	1.00	0.75	1.00	1.00	7.74	Good
P6	1.00	0.33	0.64	0.63	0.80	1.00	1.00	1.00	1.00	7.40	Good
P7	1.00	0.67	0.88	0.84	1.00	1.00	0.75	0.75	1.00	7.89	Good
P8	1.00	0.67	0.71	0.68	1.00	1.00	0.75	0.75	1.00	7.56	Good
P9	1.00	0.33	0.97	0.78	1.00	1.00	0.75	1.00	1.00	7.83	Good
P10	1.00	0.33	0.85	0.86	0.60	1.00	1.00	1.00	1.00	7.64	Good
P11	1.00	0.67	0.77	0.87	1.00	1.00	1.00	1.00	1.00	8.30	With distinction
P12	1.00	0.33	0.82	0.83	0.80	1.00	1.00	1.00	1.00	7.78	Good
P13	1.00	0.67	0.84	0.85	1.00	1.00	1.00	1.00	1.00	8.36	With distinction
P14	1.00	0.67	0.77	0.92	1.00	1.00	1.00	1.00	1.00	8.35	With distinction
Average	1.00	0.52	0.81	0.78	0.83	0.98	0.89	0.93	1.00	7.74	Good

**Table 6:** Values of the PMC matrices of the policy texts.

No.	P1			P2			P3			P4		
Matrix	1.00	0.67	0.75	1.00	0.33	0.79	1.00	0.67	0.84	1.00	0.33	0.96
	0.64	0.40	1.00	0.81	0.60	1.00	0.83	0.80	1.00	0.76	0.60	0.75
	0.75	0.75	1.00	1.00	1.00	1.00	0.75	1.00	1.00	1.00	0.75	1.00
**No.**	**P5**			**P6**			**P7**			**P8**		
Matrix	1.00	0.67	0.69	1.00	0.33	0.64	1.00	0.67	0.88	1.00	0.67	0.71
	0.63	1.00	1.00	0.63	0.80	1.00	0.84	0.80	1.00	0.68	1.00	1.00
	0.75	1.00	1.00	1.00	1.00	1.00	0.75	0.75	1.00	0.75	0.75	1.00
**No.**	**P9**			**P10**			**P11**			**P12**		
Matrix	1.00	0.33	0.97	1.00	0.33	0.85	1.00	0.67	0.77	1.00	0.33	0.82
	0.78	1.00	1.00	0.86	0.60	1.00	0.87	1.00	1.00	0.83	0.80	1.00
	0.75	1.00	1.00	1.00	1.00	1.00	1.00	1.00	1.00	1.00	1.00	1.00
**No.**	**P13**			**P14**								
Matrix	1.00	0.67	0.84	1.00	0.67	0.77						
	0.85	1.00	1.00	0.92	1.00	1.00						
	1.00	1.00	1.00	1.00	1.00	1.00						

**Fig 2:**
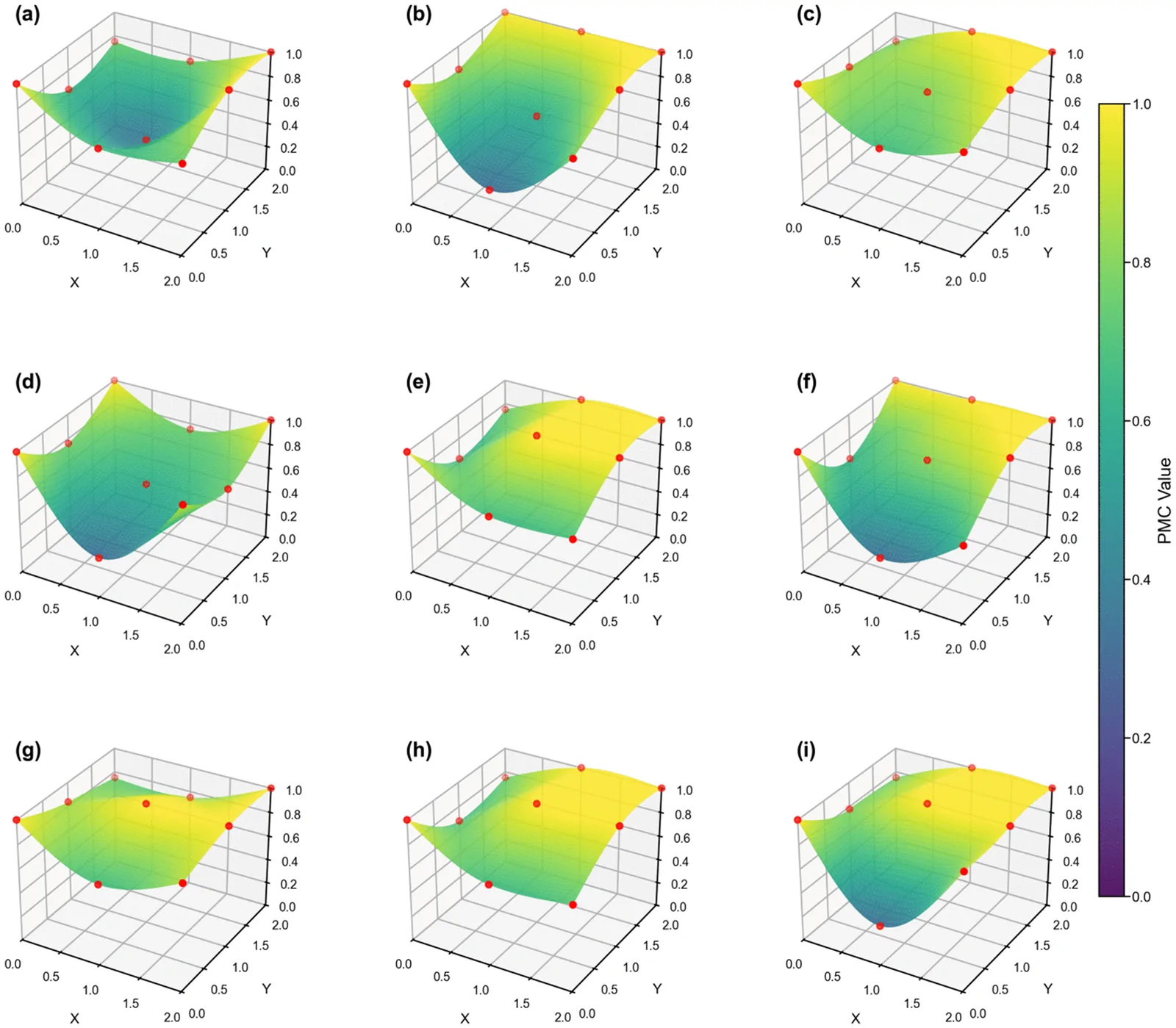
P1-P9 PMC surface graphs. (a) P1; (b) P2; (c) P3; (d) P4; (e) P5; (f) P6; (g) P7; (h) P8; (i) P9.

**Fig 3:**
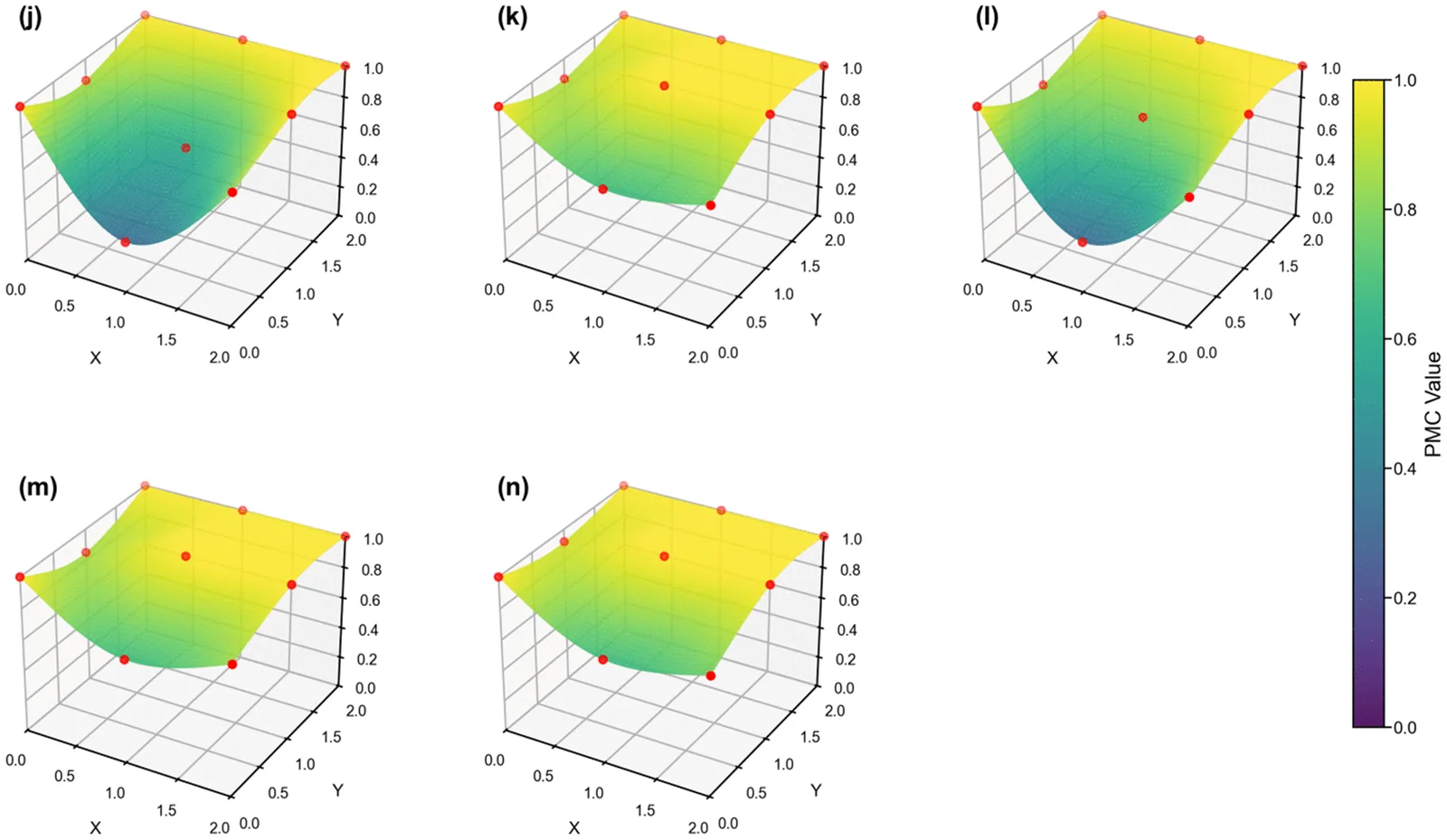
P10-P14 PMC surface graphs. (j) P10; (k) P11; (l) P12; (m) P13; (n) P14.

## 7. Outcome analysis

### 7.1. Horizontal evaluation

Based on the results presented in Table 5, among the 14 sampled policy documents, 10 were classified as“good” and 3 as “with distinction” no policies received a “fail” rating. The average score across all samples was 7.74, indicating an overall satisfactory quality of regular CHSPs. This suggests that local policy initiatives have provided strong support for regular county high school education in China. However, the absence of policies achieving a “perfect” rating as well as the fact that 6 policies scored below the average reveal room for further optimization and improvement.

The only policy that received a “pass” score was P1, issued by Jilin Province, received a PMC score of 6.96 (pass). Several indicators of this policy were lower than the average value, namely X3, X4, X5, X7, and X8, with gaps of 0.05, 0.14, 0.43, 0.14, and 0.18 respectively.In particular the weakest variables were those under X5, related to a notable lack of tools to improve leadership capabilities, individual and family education concept innovation and reform the education system. Hence, the recommended improvements should be prioritized along this path: *X*5 > *X*8 > *X*7 > *X*4 > *X*3.

P2, issued by Shenyang City, Liaoning Province, had a score of 7.53 (good), which was near the average. This policy achieved full marks in X1, X6, X7, X8, and X9, while it underperformed in X2, X3, and X5 with a gap of 0.19, 0.02, and 0.23 below the average respectively. The primary cause of the point deduction in indicator X5 can be attributed to the insufficient utilization of persuasive tools and system change mechanisms. More specifically, there is a notable absence of advocacy for the renewal of educational concepts within the context of personal and family education, as well as in policy documents aimed at systemic educational reform. This is unacceptable for a systemic problem. The recommended improvements should be prioritized along, this path: *X*2 > *X*5 > *X*3.

P3, issued by Shanxi Province, had a score of 7.88 on the PMC index (good). All the indicators scored above average except X7, which had a gap of 0.14 above average, this indicating a high quality. This gap from perfect, primarily results from the policy’s lack of detailed implementation measures. Hence, it is recommended to prioritize the refinement X7 by developing concrete operational guidelines.

P4, issued by Hulunbuir City, Inner Mongolia Autonomous Region, had a scored of 7.16 (good). In this policy indicators X2, X4, X5, X6, and X7 underperformed, with a gap of 0.19, 0.02, 0.23, 0.23, and 0.18 below average, respectively. These low scores stem from an insufficient use of persuasive policy tools and systemic reform mechanisms, issues seen also in P2. The recommended improvements should be prioritized along this path: *X*5 > *X*6 > *X*2 > *X*7 > *X*4.

P5, issued by Henan Province, had a score of 7.74 (good), in line with the average. In this policy, only X3, X4 and X7 underperformed, with a gap of 0.12, 0.15, and 0.14 from the average respectively, demonstrating relatively sound policy design. The recommended improvements should be prioritized along this path: *X*4 > *X*7 > *X*3.

P6, issued by Changsha, attained a PMC score of 7.40 (good). Indicators X2, X3, X4, and X5 had a performance below average, with a gap of 0.19, 0.17, 0.15, and 0.03 respectively. The low score in X2 is attributed to the policy’s recent enactment in 2023 with only three years of implementation history. Additionally, underperformance in X3 stems from insufficient data coverage in sub-indicators X3:1, X4:3, and X4:4. The recommended improvements prioritized along this path: *X*2 > *X*3 > *X*4 > *X*5.

P7, issued by Fujian Province, achieved a score of 7.89 (good), i.e., higher than the average. Only two indicators, X7 and X8 underperformed, with a gap of 0.14 and 0.18 from the average respectively. This result suggests that this policy demonstrates structural soundness in its core objectives, although lacking robustness in implementation safeguards. The corrective actions should be prioritized as follows: *X*8 > *X*7.

P8, issued by Hangzhou, attained a score of 7.56 (good). Indicators X3, X4, X7, and X8 underperformed, with a gap of 0.09, 0.10, 0.14, and 0.18 respectively, mirroring the safeguard deficiencies observed in P7. Notably, in sub-indicator X4:4, this policy scored only 2 out of 6 metrics, indicating inadequate support mechanisms for high school students. Improvements should be prioritized along this path: *X*8 > *X*7 > *X*4 > *X*3.

P9, issued by Guangdong Province, had a score of 7.83 (good), above the average. Only two indicators, X2 and X7, scored below average, with a gap of 0.19 and 0.14. The lower scores are mainly due to the policy’s late release in 2023 and lack of detailed implementation plans across government departments. Improvements should be prioritized as follows: *X*2 > *X*7.

P10, issued by Nanning, Guangxi Province, and P12, issued by Chengdu, Sichuan Province, achieved a score of 7.64 and 7.78, respectively (both good). Both policies underperformed in X2 and X5 indicators, with P10’s X5 gap (0.23) being significantly larger than P12’s (0.03). This is because P10 lacks initiatives to promote new educational concepts for individuals and families, while P12’s smaller X5 gap indicates that it has partially implemented them. Notably, both share an identical X2 gap (0.19), reflecting their short implementation periods (under 3 years). Improvements should be prioritized along the following path: P10:*X*5 > *X*2; P12: *X*2 > *X*5.

P11, issued by Guizhou Province, and P14, issued by Ningxia Hui Autonomous Region, were grouped for analysis as both underperformed only in the X3 indicator and exceeded the average scores for all others indicators. P11 hand a PMC score of 8.30 and P14 a score of 8.35, both attaining the distinction grade. Detailed analysis revealed that P11’s primary weakness was in sub-indicator X3:4, where it failed to meet 3 out of 6 sub-criteria. In contrast, P14 exhibited minor scattered gaps across X3 sub-indicators, contributing to its marginally higher overall score. To address these issues, P11 should focus on enhancing policy guidance for establishing structured partnerships between high-performing schools and disadvantaged institutions.

P13, issued by Gansu Province, achieved the highest PMC score of 8.36 among all 14 policies. While it demonstrated no significant weaknesses, minor limitations prevent it from reaching perfection. These include its mid-term policy framework (3–5 years) and lack of advanced reforms in classroom innovation, student development guidance, and home-school collaboration. The observed constraints suggest that medium-term policies may struggle to implement complex educational reforms, highlighting the necessity for long-term strategic planning within the CHSPs framework to enable systemic transformation.

### 7.2. Vertical evaluation

#### 7.2.1. Policy functions (X1).

With a mean score of 1.0, all the policies investigated comprehensively covered the four core policy functions (directive, supportive, encouraging, and regulatory roles). The “predictive role” and “descriptive role” were not included because Chinese government policy often places them in separate advisory reports, rather than the official policy text.

#### 7.2.2 Policy statute of limitations (X2).

The lowest mean score (0.50) for X2 stems from the national CHSPs mandate on county-level education reforms. Most local policies are aligned with the national timeline targeting completion within the 14^th^ Five-Year plan period, resulting in temporally focused, medium or short term orientations.

From the perspective of the 14^th^ Five-Year Plan as a whole, in 2021 government prioritized addressing the rapid and widespread decline of county high schools through CHSPs, mandating local governments to immediately curb the spread and exacerbation of these issues within the five-year timeframe. Consequently, local CHSPs implementation predominantly focused on short-to-medium-term policy objectives, while retaining limited long-term goals. This indicates that at that time, the central government had already recognized the systemic nature of county high schools’ challenges. In reality, the root cause of county high schools’ fragility lies in student outflow driven by urbanization [48]. To confront this systemic issue, it is necessary that CHSPs incorporate long-term countermeasures, a requirement that underscores the value of system-change policy tools in addressing structural educational disparities.

#### 7.2.3. Policy priorities (X3).

In X3:1, only eight policies addressed X3:1–1; this may potentially reflect regional disparities in the digital enrollment infrastructure. Likewise, only seven policies emphasized X3:1–5, highlighting the insufficient recognition of the role of vocational-technical education in the county education system.

Student enrollment critically reflects educational equity. Drawing on the experience of China’s online college admission system, the adoption of a unified online enrollment platform can effectively curb the enrollment “black box”, minimize human intervention, reduce operational costs, and improve administrative efficiency [49]. However, this study indicate persistent deficiencies in systemic integration and network transformation within current frameworks. While most regions in China have achieved advanced educational informatization, private high schools operating outside unified enrollment platforms undermine equity. The 2022 Notice of the Ministry of Education reiterated the urgency of establishing standardized digital admissions systems to address irregularities such as premature enrollment, over-quota recruitment, and cross-district violations [50].

The low evaluation of general-vocational integration underscores an insufficient governmental recognition of the role of vocational education in enhancing county high schools. This integration is a current trend amid economic development and industrial restructuring, an inevitable direction following the popularization of senior high school education in China, and also an essential requirement to advance the equal development of general education and vocational education [51]. One form of distinctive development of county high schools lies in the integrated development of general education and vocational education. Senior high schools adopting this integrated model are also known as comprehensive senior high schools. These schools not only offer students the opportunity to take the college entrance examination and pursue higher education, but also provide an alternative career-oriented path [52]. Therefore, the reform integrating vocational and general education serves as a new approach for differentiated development between county high schools and urban senior high schools.

#### 7.2.4. Policy object (X4).

In X4:1 low scores in cultural construction (7 policies) and safety assurance (3 policies) indicate insufficient investment in campus physical environment and risk management. School cultural development, encompassing tangible infrastructure (architecture, curricula) and intangible elements (ethos, values), critically shapes educational outcomes through material-immaterial synergy. However county high schools, burdened by academic competition and student quality disparities, often marginalize cultural construction in favor of test performance, exacerbating institutional homogenization [53]. Specialized programs (tangible classroom initiatives) must align with school cultural identity (intangible collective ethos) to drive institutional distinctiveness. For county high schools to achieve specialized development, curricula must reflect cultural uniqueness, while cultural identity requires grounding in tailored educational practices [54]. Thus,to strengthen campus culture, policy-supported institutionalization to harmonize specialized programs with school-specific cultural narratives, breaking homogeneity through integrated tangible-intangible strategies.

Furthermore, the score for campus safety was relatively low. The reason is that China has formulated dedicated policies concerning the safety of primary and secondary school campuses safety, including campus safety management [55], student accidental injury [56], bullying [57], food safety [58], mental health [59], and health management [60]. These policies have created a relatively complete system, whose scope keeps expanding together with social development [61]. Therefore, the fact that few CHSPs contain provision on campus safety can be attributed to the soundness of additional relevant policies. Moreover, county high schools are currently facing challenges in the field of teaching rather than in relation to campus safety.

In X4:4, X4:4–3 only 6 policies policies had a score under X4:3, and only 6 and 3 policies had a score under X4:4–4 and X4:4–6, respectively. In contrast all policies had a score under X4:4–1, indicating a preference for material support (e.g., funding per student) over holistic student development.

This study identified the root cause of low scores in these three indicators as systemic deficiencies in home-school collaboration policies. Specifically, the county high school education model adopts intensive exam-focused regimens with closed-campus management to compete with urban schools despite resource disadvantages [9]. This spatial segregation exacerbates family separation, particularly for rural students with migrant worker parents. While these schools prioritize academic metrics, they systematically neglect students’ holistic developmental needs. As a pivotal juncture in students’ lives, high schools bear the responsibility of providing comprehensive career and academic guidance–-a need acutely felt in county high schools, where students often lack parental or professional mentorship. Insular county societies and parents’ absence further exacerbate students’ myopic or disengaged attitudes toward career planning. A previous study revealed the following persistent issues: fragmented policy interpretation, insufficient regional coordination, narrow guidance scopes skewed toward utilitarianism, and inadequate specialization [62]. Concurrently, county and rural high school students face significant challenges in relation academic performance [63], mental health [64], and career planning capabilities [65], underscoring the urgent need for policy-driven enhancements in student guidance.

In 2023, 13 ministries jointly issued the *“Opinions on Improving Collaborative Education Mechanisms Among Schools, Families, and Society”,* aiming to build a cohesive, efficient system by 2035 [66]. Collaborative education seeks to transition family education from a “self-directed” state to a government-guided, multi-stakeholder model. However, in fact, family engagement remains as the weakest link. Previous research indicated that the educational attitudes of rural families shaped by migrant parents’ limited socioeconomic status significantly hinder children’s access to higher education as economic precarity fosters short-termism and neglect of psychological/moral development, with severity inversely correlated to socioeconomic standing [67]. This shows the need for policy interventions to cultivate sound educational philosophies in rural households, particularly through parent-oriented guidance.

#### 7.2.5. Policy tools (X5).

Eight policies had a score for X5:5; this indicates that local governments maintain a conservative stance toward the Ministry of Education’s requirement to transition from a “county-led” to a “county-city joint operation” educational governance system.

Based on the distribution of policy instruments, local policies tend to favor more conservative policy tools, aligning with the traditional reformist mindset of incremental adjustments from easy to difficult and from shallow to deep interventions. The text of the Ministry’s “14^th^ Five-Year Plan” indicates that the central government still expects local governments to actively explore systematic transformational policy instruments, thereby implementing top-down systemic reforms of the county and municipal high school education systems.

The evolution of county high schools management–-from historical county-dominated governance and operational oversight to the current exploration of a bifurcated system prioritizing prefecture-level administration for school operations and county-level management–-reflects a paradigm shift forced by institutional decline [68]. The waning trajectory of county high schools has compelled local governments to transition from fragmented governance to coordinated development frameworks. While county governments bear the responsibility to revitalize these institutions, greater accountability rests with prefecture-level authorities, necessitating cross-departmental collaboration beyond education bureaus [69].

From a macro-level perspective, China’s accelerating urbanization and increasing urban-rural integration necessitate the spatial redistribution of public services (including education) from urban centers to county-level regions [70]. Concurrently, addressing student attrition in county high schools could alleviate enrollment pressures in urban institutions, incentivizing prefecture-level governments to assume expanded operational obligations. The decline of county high schools is inextricably tied to fiscal constraints at the county level, and is exacerbated by the systematic absorption by urban high schools of top county students–-a dynamic demanding fiscal compensation mechanisms. Consequently, local governments must advance a tripartite governance model: provincial-level coordination, prefecture-level school operation, and county-level management. This institutional architecture aims to establish sustainable trusteeship and support mechanisms for county high schools. Empirical evidence demonstrates that provincial fiscal support yields more pronounced effects than municipal fiscal support in enhancing public service provision at the county level, particularly in poverty-stricken counties within mountainous regions [71]. The integration of educational funds for underdeveloped counties into provincial fiscal counterpart support mechanisms helps elevate the educational quality of local county high schools.

The strong performance observed in X6-X9 reflects the policy design capabilities of local government. As all the policies investigated are provincial/municipal-level frameworks, they intentionally allow flexibility for lower-level governments to adapt implementation plans based on local contexts. This explains why some policies show minor gaps in specific sub-indicators such as X7:2 and X8:3. Given CHSPs non-sensitive nature, X9 requires no further analysis.

## 8. Limitations

Due to technical and resource constraints, not all CHSPs were analyzed. More in detail, due to policy issuance hierarchy,this study analyzed mainly those CHSPs that have been formulated at the provincial and municipal levels, resulting in the inclusion of a lower number of CHSPs issued by county-level governments in the policy text corpus.

During the policy scoring phase, only 14 policy texts were distributed to 7 experts for evaluation, meaning inter-rater reliability could not be fully validated. However, during the subsequent proofreading process, both authors verified each individual score. The final scores were established only after confirming that there were no obvious errors or points of contention.

## 9. Policy recommendations

First, the systemic challenges of county high school decline demand a fundamental restructuring of the current CHSPs framework, which remains constrained by short-term policy cycles. To break this cycle, future policies should establish decadal strategic roadmaps centered on a holistic education reform. This requires a transition from exam-centric evaluation systems to competency-based frameworks, while creating county-level innovation hubs for pedagogical experimentation. For example, the integration of vocational-academic pathways through dual-track certification models could revitalize rural education ecosystems suffering from “cultural desertification.” These measures would help rural high schools overcome their overreliance on test-oriented drilling and genuinely implement quality-oriented education. Concurrently, CHSPs should mandate adaptive strategies for county high schools to align with the evolving national college entrance examination requirements, replacing entrenched exam-preparation models with dynamic curricula that cultivate both academic rigor and practical skills across all rural learners.

Second, a critical lever for equity lies in the digitization of enrollment systems through province-wide unified platforms. By consolidating the admission processes to public and private high schools, these platforms would not only enhance transparency and fairness–-laying the groundwork for procedural justice in education, but also enable real-time monitoring of student mobility. This data infrastructure would empower local governments to optimize resource allocation using predictive analytics, ensuring that funding decisions align with emerging educational needs. Provincial pilots in Jiangsu demonstrate that these types of digital ecosystems reduce administrative corruption by 42% while improving rural student retention rates, proving their scalability as foundational components of CHSPs 2.0.

Third, to address the urban-rural education divide, it is necessary to formulate policy interventions promoting vocational-general education convergence. This includes curriculum integration modules that blend STEM disciplines with vocational competencies, akin to Germany’s dual-system model [72], alongside certification equivalency frameworks that recognize vocational diplomas as equivalent to academic qualifications. Mandatory internship quotas—20% for urban students and 50% for rural learners–-would bridge the enterprise-academy divide while addressing rural youth’s employability gaps. These structural reforms require the allocation of 15% of provincial education budgets to incentivize school-enterprise partnerships, ensuring that vocational training programs directly respond to regional labor market demands.

Forth, it is recommended to resolve the deficit in the demand for rural student development reimagining family-school-community synergies. Governments should incentivize rural entrepreneurship through tax breaks and microloan programs to reverse rural depopulation trends, thereby increasing parental presence. Parallel investments in county-level “Family Education Hubs” staffed by trained counselors could deliver targeted parenting programs. These hubs would also serve as tele-education nodes, connecting migrant parents with school psychologists and career advisors via 5G-enabled platforms. By co-locating community resources with schools, CHSPs can transform fragmented rural education landscapes into cohesive ecosystems where academic development, family engagement, and local industry needs mutually reinforce each other.

Finally, it is imperative to expedite the reform of the education management system. While the authority of county-level governments in educational administration should be preserved, the role and influence of municipal governments in school operations should be enhanced.Regarding fiscal support, it is recommended that provincial governments establish a priority list of counties requiring targeted assistance, enabling provincial-level fiscal funds to deliver targeted support to high schools in underprivileged counties through tailored funding mechanisms.

## Supporting information

S1 Appendix Summary of CHSPs. (XLSX)

S2 Appendix Open coding of policy texts (excerpt). (XLSX)

S3 Appendix The table of evaluation criteria. (XLSX)

S4 Appendix Multi-input and output table. (XLSX)
