# Supplementary material for: A quantitative assessment of Chinese county high school policies using the PMC index model
Source: PLoS One. 2026 Nov 2;21(11):e0361807. doi: 10.1371/journal.pone.0361807 (PMC13727041; doi:10.1371/journal.pone.0361807)
Supplement: Supplementary file 1 [file pone.0361807.s005.docx]

**Reviewer Comments for Manuscript Number PONE-D-25-44663**

**Quantitative Assessment of Chinese County High School Policy via PMC Index Model**

This study addresses a relevant and important issue in the Chinese education policy context, especially given ongoing concerns about educational inequality and the decline of county high schools, as underscored by the authors. However, the paper requires major revision before it can be considered for publication for the following reasons:

1. The manuscript needs a stronger contextual and conceptual foundation. It should introduce/explain what ‘county high schools’ are, how they differ from ‘key high schools’, when did they start to decline, why their decline is important to investigate, and how the issue relates to the 14th Five-Year Plan and broader educational policy developments in China.
2. The manuscript has major language, formatting and presentation problems, e.g., grammatical errors, inconsistent capitalization, spacing problems and unclear wording. In some places, the language makes the meaning difficult to follow, such as “researchers’ subjective factors of researchers”; Some abbreviations or shortened forms are introduced too early or without proper introduction in the main text. Figures and tables also need editorial attention. For instance, consider including Figure 1 closer to the section where it is first discussed, and Figure 3 may need to be redesigned or subdivided because presenting 14 separate images with the same figure number is difficult to follow. Several table titles also need revision for clarity and concision.
3. Some methodological areas require further explanation.

- The relationship between the methods is not clearly established, i.e., between text mining, social network analysis, grounded theory and the PMC index model.
- It is not clear why grounded theory was used.
- In Table 1, it is unclear whether the dates refer to the policy issuance dates or the period when the search was conducted.
- The text mining section on pages 13-15 needs references and explanations, e.g., what is meant by statements such as “This paper is mainly used to generate semantic network graphs.”?
- Terminology needs to be consistent. For example, the use of “grounded theory” and “rooted theory”; “axial coding” and “spindle encoding” may create confusion.
- The description of PMC scoring needs clarification. For example, the sentence indicating that policies not meeting criteria receive “0 points 1” is clearly erroneous or incomplete.
- The steps of the PMC model could be explained for readers who are not familiar with the method.
- Were the high-frequency words in Table 2 translated from Chinese?
- The manuscript states that Table 3 shows 43 initial categories, but the table appears to show 11 examples.
- The saturation test section is particularly unclear and should be rewritten.
- The selection of the 14 policies needs more detail to justify representativeness and reproducibility. What is the full eligible pool and why can the resulting 14-policy sample be considered “representative” or “typical” of the broader 55-policy corpus?
- No details of the experts were provided at all.

4. Several key sections are under-referenced. The introduction contains very few references despite making broad claims about the significance of county high schools, their decline, and policy developments. Similarly, the methods section, especially the text mining discussion, lacks sufficient methodological support. The justification for selecting the PMC index model also needs more explicit engagement with the literature. For example, when the authors state that the PMC index model has “gained increasing recognition” in public policy research, it is not clear whether this means recognition mainly in the Chinese context or more globally. The manuscript would benefit from more careful sourcing in the introduction, methods, discussion, and final policy recommendation sections.
5. Outcome analysis section lacks citations or clarity.

- Some wording is also unclear, for example “this study will no longer be conducted in depth alone” and “proves conducive to improving…”.
- Some claims and statements such as those concerning ministry notices, policy urgency, and the effectiveness of certain policy directions require citation.
- Why do not some policies appear to receive improvement priority tracks e.g., P12?

6. The Conclusion and policy Proposal sections need stronger support and better organization.

- Reference to P11 is missing in the conclusion, ‘only three achieving distinction’.
- Section 9 presents contextual information and policy background that would be more useful if introduced earlier in the paper.
- Is this section intended as recommendations for improving existing policy, or as a proposal for a new policy framework? That distinction is currently not clear.
- Comparisons with countries such as Germany and Brazil also need stronger justification and citations.

Minor and editorial comments

1. Please define all key terms clearly at first use in the main text, even if they already appear in the abstract.
2. Reformat tables to improve readability, especially where categories and subcategories are visually difficult to follow. You may consider adding lines to divide categories.
3. Revize repetitive or awkward table titles, for example ‘Table 6 The table of evaluation criteria in detail’ and Table 8 ‘Evaluation evaluation of’, and similar constructions.
4. Clarify expressions such as “previous article,” “assignment,” etc., which are currently unclear.
5. Correct typographical and grammatical issues throughout, including examples such as “it was not collect all CHSCs.” and
