# Supplementary material for: A quantitative assessment of Chinese county high school policies using the PMC index model
Source: PLoS One. 2026 Nov 2;21(11):e0361807. doi: 10.1371/journal.pone.0361807 (PMC13727041; doi:10.1371/journal.pone.0361807)
Supplement: Supplementary file 2 [file pone.0361807.s007.docx]

#### **Response to Reviewers**

Reviewer #2: The manuscript addresses a timely and important topic—Quantitative Assessment of Chinese County High School Policy via the PMC Index Model. The focus on policy evaluation at the county high school level is both relevant and valuable, and the authors should be commended for engaging with this core issue.

At the same time, several aspects of the manuscript could be improved to enhance clarity, rigor, and overall quality.

Comment 1. Technical and Formatting Issues

There are recurring technical inconsistencies throughout the manuscript. Several sentences lack proper spacing, which affects readability. In addition, there are inconsistencies in capitalization (e.g., “On the other side, With the end of the…” on p. 9) and formatting (e.g., “table” vs. “Table”). Long passages could also be divided into shorter paragraphs to improve readability and structure.

Response:Thank you for your correction. We have revised these issues in the manuscript.

Comment 2. Language and Grammar

A number of sentences are grammatically incomplete or lack verbs, which makes them difficult to understand. For example:

“Local governments' conservative stance toward policy evaluation and systemic reforms in education.” (p. 8)

“Increase investment in career guidance and mental health education for students and product more programs to process to parents and communities join education.” (p. 8)

“County high schools are constantly mentioned in various local policies at all levels.” (p. 9)

A thorough language revision is strongly recommended.

Response:Thank you for pointing out the issues. We have corrected these errors in the manuscript and reviewed its entire content.

Comment 3. Keywords Formatting

The keywords section requires revision, as the font differs from the rest of the manuscript and spacing between words is inconsistent.

Response:Thank you for your correction. We have revised the manuscript accordingly.

Comment 4. Introduction and Use of References

The introduction would benefit from stronger support through references. Several claims are presented without citation (e.g., p. 2: “It is of great significance for the construction of modern…”; p. 3: “According to 2023 statistics from the Ministry of Education”). Please ensure that all factual statements are supported by appropriate sources.

Response:Thank you for your suggestions. We have removed the uncited and meaningless claims from the manuscript and added citations for data sourced from the Ministry of Education.

Comment 5. Research Design

The research design requires further clarification and justification:

（1）Please justify the selection of Peking University as a case or source.

（2）The conceptualization of “policy” appears somewhat superficial and should be strengthened to reflect its nature as a governmental and institutional document.

（3）The rationale for the selected timeframe (2021–2024) is not explained and should be justified.

（4）Some tables (e.g., Table 1) could be moved to the Appendix, as they currently take up substantial space.

Response:（1）This was a mistake on our part. Peking University itself is not a case in point. Instead, it refers to a policy document query website with the domain name https://www.pkulaw.com. Founded with the support of Peking University, this platform is a commonly used tool for policy researchers in China to look up policy documents. It allows users to download policy documents in various formats, which enables us to employ other programming tools for word segmentation, statistical analysis and coding of policy texts in the text mining sections of our manuscript. We have added relevant explanations for this part in the manuscript.

（2）Thank you for your suggestions. We have further clarified in the relevant paragraphs of the article that the research object of this paper is policy texts.
（3）The reason for selecting 2021-2024 as the time frame is that the 14th Five-Year Plan issued by the central government in 2021 served as the blueprint for local governments to formulate their own CHPs. Consequently, local governments could only release their CHPs after 2021. Meanwhile, the 14th Five-Year Plan covers a five-year period, and local governments need at least one year to fulfill the policy goals set out in their CHPs. Therefore, the latest release date of the CHPs cannot be later than 2024. This content has been added to the manuscript.
（4）We have adjusted the position of Table 1 as you requested and moved it to S1 Table in the supporting information.

Comment 6. Redundancy (SNA Section)

The section on Social Network Analysis (SNA) appears partially redundant, as key elements are already introduced in the research design. Consider removing or streamlining this section to avoid duplication.

Response:Thank you for your suggestions. We have simplified this section of the manuscript while retaining the word frequency statistics. The reason for keeping the word frequency statistics is that after we identified X3, X4 and X6, we needed to clarify the specific contents of other CHPs and which indicators they respectively belong to. These serve as the secondary indicators of X3, X4 and X6, while the tertiary indicators are derived from GT.

Comment 7. Figures and Visuals

Some figures are not well integrated into the text. For instance, “The specific process is shown in Fig. 1” is mentioned in the main text, but the figure is placed in the Appendix. Either relocate the figure to the main text or clearly indicate that it can be found in the Appendix.

Response:Thank you for your suggestions. We have made revisions to the manuscript.

Comment 8. Policy Language and Translation

The manuscript does not specify the language of the policy documents analyzed. If the policies were originally in Chinese, please clarify:

how the translation was conducted

how accuracy and consistency were ensured

Response:We have reannotated the footnotes for Table 1 in the manuscript to indicate that the original versions of these policy documents are in Chinese. Most of these documents have official English translations available on relevant official websites, while a small number were translated by ourselves. To ensure translation quality, we have proofread all translations in the manuscript once again.

Comment 9. Use of Software (NVivo)

Although NVivo 12 Plus is mentioned, it is not sufficiently described in the research design. Please clarify:

how the software was used

why this specific tool was chosen

Response:When conducting coding with NVivo12plus, we compile all original texts into a single file and import it. The software can recognize the text within the file, allowing us to label and categorize each text segment in the program. We choose NVivo12plus for two main reasons. Firstly, it boasts high compatibility with Chinese texts and runs stably, without crashing even when processing large volumes of text.

Comment 10. Coding and Reliability

The “Open coding” section lacks important methodological details:

Who conducted the coding?

How was inter-coder reliability ensured?

Response:For the coding work, the corresponding author formulated the guidelines for text coding, while the first author and another doctoral student conducted the specific coding. To ensure the reliability of the results, we randomly selected 10 texts to test reliability using Cohen’s Kappa coefficient, which was calculated as 0.78. Relevant details have been added to the manuscript.

Comment 11. Saturation Test

The first paragraph of the “Saturation test” section requires revision. Currently, the text consists of fragmented sentences, making it difficult to follow the argument.

Response:Thank you for your suggestions. We have revised this paragraph and added relevant technical details. To verify that no new categories emerged after coding the 55 policy documents, we selected an additional three policy files and conducted verification following the three-round coding procedures. No new categories were identified in the results.

Comment 12. Variable Definition (Table 4)

Response:Among the primary variables, X3, X4 and X6 are directly derived from the "14th Five-Year Plan". Issued by the Ministry of Education, this policy document serves as the blueprint for local governments to formulate CHPs, and it clearly specifies policy objectives, policy priorities, and policy guarantees. The remaining primary variables are adopted from those established by other scholars. The detailed information is presented in Table 4 with corresponding literature citations attached.

It is unclear how Level 1 variables were defined. This should be explicitly explained, either in the text or in a note accompanying the table.

Comment 13. Multi-input-output Table

The description of the multi-input-output table appears unnecessary and could be shortened. Table 6 could also be moved to the Appendix. Additionally, the statement on p. 32 (“the results formed were cross-verified”) requires clarification how this verification was conducted.

Response:We have simplified the description of the Multi-input-output Table and moved Table 6 to the appendix. Meanwhile, we have rewritten the scoring process for Table 7. We selected seven graduate students as targeted samples, among whom two specialize in public policy and five focus on educational policy. All raters received systematic training on the standardized scoring criteria presented in Table 6 and took part in trial scoring to ensure a consistent understanding of the evaluation standards. Each rater independently scored two different policy documents in accordance with the established scoring scale. Since the policy texts assigned to different raters did not overlap, inter-rater reliability indicators such as Cohen's Kappa coefficient were not calculated in this study.

Comment 14. PMC Surface Construction

The description of the PMC surface could be simplified and presented more concisely, ideally in one clear sentence.

Response:Thank you for your suggestions. We have streamlined the discussion of this section while retaining the core viewpoints.

Comment 15. Limitations and Future Research

Consider moving the limitations section before the conclusion. It would also strengthen the manuscript to include directions for future research.

Response:We have made adjustments as you suggested.

Comment 16. Policy Proposals

The policy proposal section is currently overly descriptive. Presenting the recommendations in bullet points would improve clarity and readability.

Response:Thank you for your suggestions. We have simplified part of the content for easier reading.

Reviewer3#This study addresses a relevant and important issue in the Chinese education policy context, especially given ongoing concerns about educational inequality and the decline of county high schools, as underscored by the authors. However, the paper requires major revision before it can be considered for publication for the following reasons:

1. The manuscript needs a stronger contextual and conceptual foundation. It should introduce/explain what ‘county high schools’ are, how they differ from ‘key high schools’, when did they start to decline, why their decline is important to investigate, and how the issue relates to the 14th Five-Year Plan and broader educational policy developments in China.

Response:County high schools are senior high schools run by county-level governments that provide general education. They are actually a product of the key school policy introduced in the 1950s and are essentially key senior high schools. [5][6] However, over the past decade or so, the in-depth integration of urban middle schools with social capital, along with intentional or unintentional policy preferences of local governments, has given rise to the emergence of "super high schools" in urban areas. [8] Boasting favorable geographical locations and abundant funds, these "super high schools" maintain their academic performance by poaching outstanding teachers and students from county high schools. [9] The Ministry of Education has noticed these issues and issued national policies to halt the continuous decline of county high schools. The *Outline of the People’s Republic of China 14th Five-Year Plan for National Economic and Social Development and Long-Range Objectives for 2035* does not directly mention county high schools, but sets requirements for the popularization of senior high school education. The core policy requirements come from the *Outline of Plan for Building China into a Leading Country in Education (2024-2025)*, which explicitly puts forward the goal of "boosting the development of senior high schools in county-level areas". There are indeed deficiencies in the manuscript regarding the value and significance of researching this issue, and we have supplemented relevant content in the revised version of the manuscript.

2. The manuscript has major language, formatting and presentation problems, e.g., grammatical errors, inconsistent capitalization, spacing problems and unclear wording. In some places, the language makes the meaning difficult to follow, such as “researchers’ subjective factors of researchers”; Some abbreviations or shortened forms are introduced too early or without proper introduction in the main text. Figures and tables also need editorial attention. For instance, consider including Figure 1 closer to the section where it is first discussed, and Figure 3 may need to be redesigned or subdivided because presenting 14 separate images with the same figure number is difficult to follow. Several table titles also need revision for clarity and concision.

Response:Thank you for pointing out the issues. We have revised them in the manuscript.

3. Some methodological areas require further explanation.
(1) The relationship between the methods is not clearly established, i.e., between text mining, social network analysis, grounded theory and the PMC index model.
(2) It is not clear why grounded theory was used.
(3) In Table 1, it is unclear whether the dates refer to the policy issuance dates or the period when the search was conducted.
(4) The text mining section on pages 13-15 needs references and explanations, e.g., what is meant by statements such as “This paper is mainly used to generate semantic network graphs.”?
(5) Terminology needs to be consistent. For example, the use of “grounded theory” and “rooted theory”; “axial coding” and “spindle encoding” may create confusion.
(6) The description of PMC scoring needs clarification. For example, the sentence indicating that policies not meeting criteria receive “0 points 1” is clearly erroneous or incomplete.
(7) The steps of the PMC model could be explained for readers who are not familiar with the method.
(8) Were the high-frequency words in Table 2 translated from Chinese?
(9) The manuscript states that Table 3 shows 43 initial categories, but the table appears to show 11 examples.
(10) The saturation test section is particularly unclear and should be rewritten.
(11) The selection of the 14 policies needs more detail to justify representativeness and reproducibility. What is the full eligible pool and why can the resulting 14-policy sample be considered “representative” or “typical” of the broader 55-policy corpus?
(12) No details of the experts were provided at all.

Response:

3.1Text mining is a research method for constructing the indicators in the PMC index model. This method encompasses social network analysis (which we have simplified to word frequency statistics as suggested by Reviewer 2) and grounded theory. The indicator system of the PMC index model consists of two primary indicators: policy priorities and policy targets. The secondary indicators under them are derived from partial results of word frequency statistics, while the tertiary indicators included in the secondary indicators are obtained through grounded theory.

3.2The reason for adopting grounded theory is that it is the most direct and reliable method for constructing the indicator system of the PMC index model. Previous studies generally either directly adopted the indicator system established by Estrada[16], or constructed a new one by combining word frequency statistics with research findings from other scholars. The most prominent flaw of these approaches is that the indicators they created lack clear theoretical basis and are not tailored to the policy texts under measurement. Hence, the scientificity and applicability of their indicator systems remain questionable. To maximize the scientific validity of the indicators, we employed grounded theory to conduct an in-depth analysis of policy texts, so as to clarify the origins of each indicator as thoroughly as possible.

3.3We have redesigned Table 1 and added the release dates of the policies. Following the suggestions from Reviewer 2, we have simplified this section to word frequency statistics. We adopt the word frequency statistics method to construct the first-level indicators. Relevant citations have been added to the manuscript.

3.4We have adopted the suggestions put forward by Reviewer 2, and this part has been simplified to word frequency statistics. We adopt the word frequency statistics method to construct the secondary indicators. Relevant citations have been supplemented in the manuscript.

3.5Thank you for pointing out the issues. We have corrected these errors in the manuscript.

3.6We have redesigned the statements in the evaluation criteria to ensure they are as clear and easy to read as possible. To enhance overall readability, we have moved them to the supporting information section.

3.7We have added a detailed introduction to the procedures after "The Research Process".

3.8 All words in Table 1: Top 50 high-frequency words in policy texts are translated from Chinese.

3.9 We have further refined this section and clarified that there are 48 initial categories. Both these initial categories and their corresponding paragraph examples from the original policy text have been redesigned; see Appendix S2 in the supporting information for details.

3.10 I have rewritten this section.After coding the original 55 CHSP documents, this study performed a theoretical saturation test. Three purposively sampled county, provincial, and municipal policies were analyzed, with only segments explicitly mentioning "county high school" coded. The two authors applied consistent coding procedures, resolving discrepancies through discussion. No new concepts or categories emerged, confirming full theoretical saturation.

3.11We selected two provinces or cities from each of the seven major regions, namely Northeast China, North China, Central China, East China, South China, Southwest China and Northwest China, to ensure that the selected policy documents cover the whole country. The two provinces or cities chosen from each region are relatively developed in economy and society with a high degree of urbanization. Given their high urbanization rates, the problem of declining county-level development is more prominent in these areas.[26] Therefore, the selected policy documents are universal and representative.

3.12 Two of the experts involved in the scoring hold master's degrees in Public Administration, and another five are master's students majoring in Educational Policy. Each of them scored two policy documents. Since the parts they were responsible for did not overlap, Cohen's Kappa coefficient was not calculated.

4. Several key sections are under-referenced. The introduction contains very few references despite making broad claims about the significance of county high schools, their decline, and policy developments. Similarly, the methods section, especially the text mining discussion, lacks sufficient methodological support. The justification for selecting the PMC index model also needs more explicit engagement with the literature. For example, when the authors state that the PMC index model has “gained increasing recognition” in public policy research, it is not clear whether this means recognition mainly in the Chinese context or more globally. The manuscript would benefit from more careful sourcing in the introduction, methods, discussion, and final policy recommendation sections.

Response:Thank you for pointing out the issues. We have added a large number of references to the manuscript. The application of the PMC model in public policy research is limited to China, and we have also supplemented relevant references in the manuscript to verify this point.

5. Outcome analysis section lacks citations or clarity.

- Some wording is also unclear, for example “this study will no longer be conducted in depth alone” and “proves conducive to improving…”.
- Some claims and statements such as those concerning ministry notices, policy urgency, and the effectiveness of certain policy directions require citation.
- Why do not some policies appear to receive improvement priority tracks e.g., P12?

Response:We have revised these grammatical errors in the manuscript and added literature citations for all the policies mentioned therein. We combined P12 and P10 into one paragraph for analysis. This is because their PMC scores are close to each other, and neither of them meets the standards for the two indicators X2 and X5. Therefore, we believe they share the same issues, which is why we analyze them together in a single paragraph.

6. The Conclusion and policy Proposal sections need stronger support and better organization.

- Reference to P11 is missing in the conclusion, ‘only three achieving distinction’.
- Section 9 presents contextual information and policy background that would be more useful if introduced earlier in the paper.
- Is this section intended as recommendations for improving existing policy, or as a proposal for a new policy framework? That distinction is currently not clear.
- Comparisons with countries such as Germany and Brazil also need stronger justification and citations.

Response: We have added the missing content on P11 in the manuscript. Section 9 in the manuscript serves as a summary of the previous analysis of 14 policy documents using the PMC index model, aiming to help other researchers working on related topics quickly locate the conclusions. We have also supplemented the missing references concerning Germany and Brazil in the manuscript.

Minor and editorial comments

1. Please define all key terms clearly at first use in the main text, even if they already appear in the abstract.
2. Reformat tables to improve readability, especially where categories and subcategories are visually difficult to follow. You may consider adding lines to divide categories.
3. Revize repetitive or awkward table titles, for example ‘Table 6 The table of evaluation criteria in detail’ and Table 8 ‘Evaluation evaluation of’, and similar constructions.
4. Clarify expressions such as “previous article,” “assignment,” etc., which are currently unclear.
5. Correct typographical and grammatical issues throughout, including examples such as “it was not collect all CHSCs.” and

Response:Thank you for your suggestions. We have revised the manuscript accordingly.
