# Supplementary material for: A quantitative assessment of Chinese county high school policies using the PMC index model
Source: PLoS One. 2026 Nov 2;21(11):e0361807. doi: 10.1371/journal.pone.0361807 (PMC13727041; doi:10.1371/journal.pone.0361807)
Supplement: Supplementary file 3 [file pone.0361807.s008.docx]

**Reviewer Comments for Manuscript Number PONE-D-25-44663**

Response to Reviewer #3:

We have revised the errors listed in 1.and 2. in the manuscript.

3. The issue you raised has been added to the limitations section.

4. Regarding the problems with Table 1 you pointed out, the reasons are as follows. First, some Chinese policy terms have similar meanings after being translated into English, yet they refer to different concepts in the original policy context, so they are retained as separate entries, such as S10 & 30. We use "Managed support" to describe China’s practice of pairing high-quality schools with underdeveloped schools via administrative orders. This pairing practice includes comprehensive guidance and intervention provided by high-quality schools in teaching and administration of underperforming schools, as well as scenarios where high-quality schools directly send management teams to take charge of underdeveloped schools. In policy documents, "support" frequently refers to fiscal support provided by higher-level governments to lower-level governments, or permission and incentives for lower-level governments to launch new initiatives. Although certain English words and phrases appear semantically similar, our research based on Chinese policy texts reveals that their connotations in Chinese do not fully align with their definitions in English. To ensure rigor, we have replaced "Managed support" with "Entrusted management assistance", which also addresses the concerns about S48 & 50. For the error concerning S15 & 39, we have revised Entry 39 to "Instructional research" and updated Table 2 accordingly.

We have replaced "words" with "term" in the title of Table 1. Since the original meanings of certain Chinese vocabulary cannot be accurately conveyed by single English words, phrases are adopted for expression.

5. We have revised X4:2-3 to "teacher personnel management". We categorize collaboration education (X4:4-6) under the "students" category for the following reasons. We refer to the collaborative education involving society, families and schools as "collaboration education". In this collaborative model, students act as the core and primary targets. Therefore, in line with our framework of categorizing policy subjects based on educational stakeholders, we believe it is reasonable to classify collaboration education under the student dimension. Furthermore, the primary objective of collaboration education in China is to improve students’ comprehensive abilities and qualities and cultivate their moral values, rather than boosting academic performance. In the Chinese context, teaching quality and teaching reform mainly aim to enhance students’ academic capabilities. At present, the evaluation of teaching quality in China still largely relies on students’ examination scores. Hence, we argue that classifying collaboration education under teaching quality and teaching reform fails to reflect the original intention of policy makers.

For readers’ convenience, we have added an explanation of collaboration education in the notes of Table 2.
