# Supplementary material for: A quantitative assessment of Chinese county high school policies using the PMC index model
Source: PLoS One. 2026 Nov 2;21(11):e0361807. doi: 10.1371/journal.pone.0361807 (PMC13727041; doi:10.1371/journal.pone.0361807)
Supplement: Supplementary file 4 [file pone.0361807.s009.docx]

Dear reviewer#3:

We sincerely appreciate your careful review of our paper. We have not only addressed the issues you pointed out but also fixed other problems.

1. We have already conducted a full-text proofread.

2.1 We have corrected the citation for the 13th reference entry to the correct format and eliminated duplicate citations.。

2.2 We have revised the errors in Section 7.1 by correcting the number of policies scoring below 7.74 to **six**, and updating the average score to 7.74.

2.3 We have corrected errors in the abstract by uniformly replacing "credit" with "good";

2.4 We have also corrected the spelling of certain names.

Wish you all the best in your work and life.
